# The Marine Natural Product Manzamine A Targets Vacuolar ATPases and Inhibits Autophagy in Pancreatic Cancer Cells

**DOI:** 10.3390/md11093500

**Published:** 2013-09-17

**Authors:** Georgios Kallifatidis, Dominic Hoepfner, Tiphaine Jaeg, Esther A. Guzmán, Amy E. Wright

**Affiliations:** 1 Marine Biomedical and Biotechnology Research Program, Harbor Branch Oceanographic Institute, Florida Atlantic University, 5600 US 1 North, Fort Pierce, FL 34946, USA; E-Mails: geokallifa@aol.com (G.K.); awrigh33@hboi.fau.edu (A.E.W.); 2 Novartis Institutes for BioMedical Research, Developmental & Molecular Pathways, Novartis Pharma AG, WSJ-355.1.051.21, Fabrikstrasse 22, Basel CH-4056, Switzerland; E-Mails: dominic.hoepfner@novartis.com (D.H.); tiphaine.jaeg@wanadoo.fr (T.J.)

**Keywords:** manzamine A, vacuolar ATPase, lysosome, autophagy, pancreatic cancer

## Abstract

Manzamine A, a member of the manzamine alkaloids, was originally isolated from marine sponges of the genus Haliclona. It was recently shown to have activity against pancreatic cancer cells, but the precise mechanism of action remained unclear. To further our understanding of the mechanism of action of manzamine A, chemogenomic profiling in the yeast *S. cerevisiae* was performed, suggesting that manzamine A is an uncoupler of vacuolar ATPases. Fluorescence microscopy confirmed this effect on yeast vacuoles, where manzamine A produced a phenotype very similar to that of the established v-ATPase inhibitor bafilomycin A1. In pancreatic cancer cells, 10 µM manzamine A affected vacuolar ATPase activity and significantly increased the level of autophagosome marker LC3-II and p62/SQSTM1 as observed by western blot analysis. Treatment with manzamine A in combination with bafilomycin A1 (inhibitor of autophagosome-lysosome fusion) did not change the levels of LC3-II when compared to cells treated with bafilomycin A1 alone, suggesting that manzamine A is a potential inhibitor of autophagy by preventing autophagosome turnover. As autophagy is essential for pancreatic tumor growth, blocking this pathway with manzamine A suggests a promising strategy for the treatment of pancreatic cancer.

## 1. Introduction

Tumor cells often reside in a hypoxic microenviroment and accumulating evidence suggests that hypoxia and the resulting increased tumor acidity are involved in cancer progression and tumor resistance to chemotherapy [1,2]. Tumor cells have developed compensatory mechanisms that confer survival and growth advantages under the unfavorable acidic conditions in the tumor microenviroment [1]. A key role with regards to this adaptation is attributed to the vacuolar ATPase (v-ATPase) [1,2]. V-ATPases are enzymes that utilize energy freed by the hydrolysis of ATP to pump protons from the cytoplasm to the lumen of intracellular compartments such as lysosomes [3,4], thus maintaining an alkaline pH in the cytoplasm. This mechanism helps tumor cells to free themselves from H+ ions that otherwise may accumulate in the cytoplasm and stimulate lytic enzymes that are active at low pH [2]. In some malignant cells, including pancreatic cancer cells, v-ATPases are also located in the plasma membrane where they pump protons from the cytoplasm to the exterior [1]. This creates an acidic tumor microenvironment which is of importance for cancer initiation and progression and thus v-ATPases are considered potential therapeutic targets in cancer [2].

V-ATPases also play a role in chemoresistance of cancer cells. One mechanism for resistance to therapy is the neutralization of weakly basic drugs by the acidic tumor microenvironment [2,4]. Lysosomes are the garbage disposal system of a cell and play a central role in autophagy. The localization of v-ATPases in the membranes of lysosomes reveals a potential role in activation of lysosomal enzymes which are only functional at low pH [5]. The low intralysosomal pH which is attributed to the v-ATPases activates resident hydrolases responsible for the degradation of various cargos delivered by autophagic processes [6]. Autophagy is a proteolytic process activated upon cell starvation to recycle cellular organelles for new protein synthesis and/or energy production. In established hypoxic tumors, such as pancreatic ductal adenocarcinoma (PDAC), autophagy is up-regulated and tumor cells use its catabolic function in order to tolerate stress [7]. In pancreatic cancer cells, transformation by oncogenic K-Ras may induce basal autophagy, which is required in these cells to maintain energy balance for tumor growth [7]. Recently macropinocytosis of protein followed by lysosomal degradation has been demonstrated to provide amino acids such as glutamine which are required for growth in Ras-transformed cells [8]. Thus, drugs that inactivate autophagic proteolysis may have a unique potential to treat cancers such PDAC [7], or to sensitize them for chemotherapeutic treatments.

Manzamine A is an alkaloid isolated from sponges of the genera *Haliclona* sp., *Xestospongia* sp. and *Pellina* sp. [9,10,11]. It has been reported to have anti-tumor, insecticidal, antibacterial, anti-malarial, and anti-inflammatory activities [12,13,14,15,16]. We recently showed that manzamine A decreased cell dissociation, abrogated cell migration and sensitized AsPC-1 pancreatic adenocarcinoma cells towards TRAIL induced apoptosis [17]. To further our understanding of the mechanism of action of manzamine A, chemogenomic profiling in the yeast *S. cerevisiae* was performed resulting in a correlation of manzamine A to known uncouplers of vacuolar ATPases. The present study aimed to further elucidate the mechanism of action of manzamine A and focused our research on the effect of manzamine A on v-ATPases and autophagy in pancreatic cancer cells.

## 2. Results and Discussion

### 2.1. Chemogenomic Profiling Suggests That Manzamine A Interferes with V-ATPases

**Figure 1:**
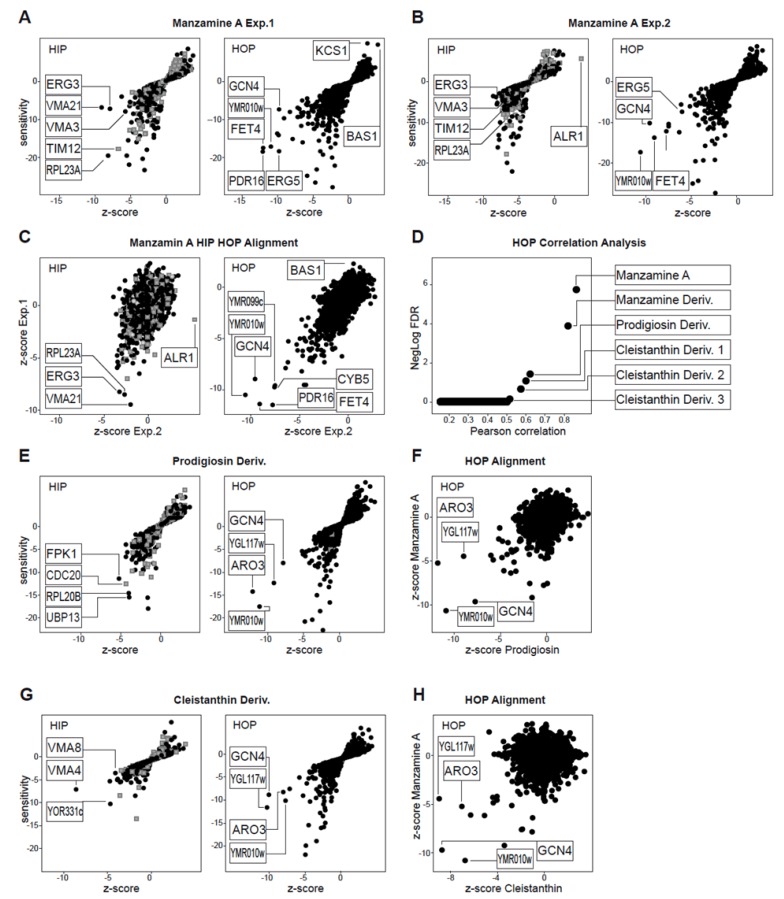
Manzamine A HIP HOP Profiling. (**A**,**B**) HIP HOP profiling of manzamine A in two individual experiments. Both independent HOP experiments identified common hits that are relevant based on *z*-score (*z*-score < −7); (**C**) *z*-score alignment of the two individual manzamine A experiments; (**D**) For reasons explained in the results section individual gene functions of hits were not analyzed in detail but the manzamine HOP profile was used in a correlation analysis against a database of HOP profiles of 2500 diverse chemical structures. This revealed correlation with other manzamine A experiments, metacycloprodigiosin and a cluster of cleistanthin substances. (**E**) HIP HOP profiles of metacycloprodigiosin. (**F**) HOP profile alignment of manzamine A and metacyloprodigiosin. (**G**) HIP HOP profile of the best correlating cleistanthin compound. (**H**) HOP profile alignment of manzamine A and cleistanthin. HIP HOP profiles are plotted by sensitivity and relevance (*z*-score). Grey boxes represent strains with deletions in essential genes, black dots strains with deletions in non-essential genes.

We first evaluated whether Manzamine A was active against the yeast *S. cerevisiae* as the availability of *g*enome-wide deletion collections for this eukaryotic model system enable powerful genetic assays to elucidate the mechanism of action for bioactive molecules [18]. At 10 μM, manzamine A inhibited growth by 30%, and was tested at this concentration by chemogenomic profiling using the heterozygous and homozygous deletion collections. The basic concept is that the haploinsufficiency profiling assay (HIP) identifies genes where one functional copy, as compared to two, confers hypersensitivity to the compound. This indicates pathways directly affected by the compound. The homozygous profiling assay (HOP), where both gene copies are deleted, identifies pathways that are compensating (synthetically lethal) for those directly affected by the compound [19]. Manzamine A was tested in two independent experiments and the HIP HOP profiles were generated (Figure 1A,B). Both HIP HOP experiments showed a good correlation when plotted against each other (Figure 1C) suggesting the manzamine A response was robust and reproducible. However, in the HIP experiment only sensitive strains of low relevance were detected, as defined by calculation of a z-score across the HIP HOP profiles of 2500 diverse compounds [20]. A z-score within a range of +/−7 indicates a strain that responds to many diverse chemotypes and is thus, not relevent for interpretation. A flat HIP profile may indicate the primary target is not a protein, or is not conserved in yeast. In contrast, the HOP profiles revealed reproducible sensitive hits with a z-score < −7 suggesting they are relevant and specific for the manzamine A mechanism of action. However, due to the synthetic lethality concept of the HOP experiments it can be challenging to interpret individual strain sensitivities. Thus, we used a comparative approach and correlated an individual manzamine A HOP profile to our database of over 2500 HOP profiles for diverse chemical substances. The best correlation was to the HOP profile for the second manzamine A experiment, followed by a different manzamine derivative, underlining the validity of the applied approach. Interestingly, the next best correlating compounds were metacycloprodigiosin and a set of cleistanthin derivatives. Analysis of the HIP HOP profiles and direct alignment against the manzamine A HOP profile revealed a conserved set of sensitive HOP strains (Figure 1E–H). Prodigiosins have been established as uncouplers of H+ ATPases including the v-ATPase [21,22,23], uncoupling ATP hydrolysis from proton transport.The identified cleistanthins have been experimentally validated to affect vacuolar morphology and acidification of vacuoles in *S. cerevisiae* similar to bafilomycin A1, an established v-ATPase inhibitor [20]. In addition, cleistanthin derivatives share a structural core also found in diphyllin, a natural product reported to potently inhibit v-ATPase [24]. Thus, the HIP HOP profiling provides two key observations: (1) the absence of reproducible, relevant HIP hits suggests a non-protein target effect, and (2) the reproducible HOP profile correlates with established v-ATPase uncouplers. Taken together, we conclude that in yeast, manzamine A likely acts as an uncoupler of v-ATPase activity.

### 2.2. Effects of Manzamine A on Yeast Vacuolar Morphology and Proton Gradient

**Figure 2:**
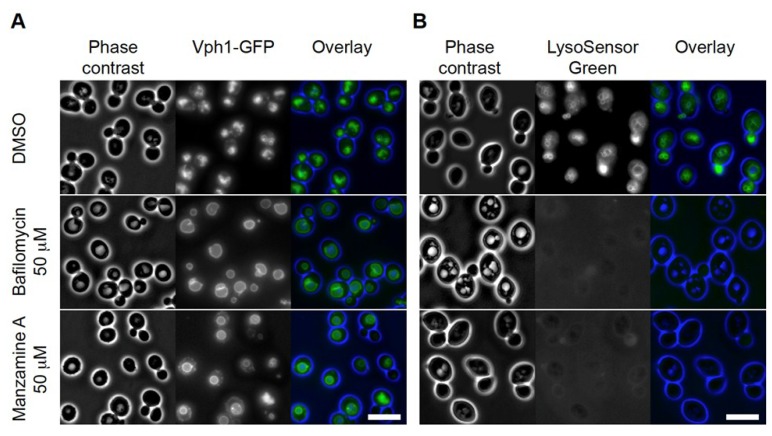
Manzamine A affects vacuolar morphology and acidification in yeast, similar to bafilomycin. (**A**) Vacuolar morphology analysis using Vph1-GFP (a v-ATPase V0 domain) as marker. DMSO treated cells showed clusters of small vacuoles. In bafilomycin A1 treated cells one large vacuole was detected in almost all cells. Manzamine A treated cells displayed a few enlarged vacuoles similar to the situation observed in bafilomycin A1 treated cells. (**B**) Vacuolar acidification analysis using LysoSensor Green as marker. DMSO treated cells show staining of the vacuolar membranes. Treatment of cells with bafilomycin A1 or manzamine A results in abolishment of detectable vacuolar staining. Size bar represents 5 µM.

Next, we tested if effects on yeast vacuoles could be validated by a different approach. Inhibition of *S. cerevisiae* v-ATPase and modulating the proton gradient causes an increase in vacuole size and loss of luminal acidification, which can be assessed by fluorescence microscopy with (i) a vacuolar limiting membrane marker, Vph1-GFP, and (ii) the loss of LysoSensor fluorescence respectively [25,26]. We incubated Vph1-GFP labeled cells for 60 min with 5 × the IC_30_ concentration of the established v-ATPase inhibitor and manzamine A and analyzed the results in comparison to DMSO treated cells. Whereas DMSO treated cells showed clusters of 2–6 small vacuoles, bafilomycin A1 treatment resulted in formation of primarily a single large vacuole, (Figure 2A). In cells treated with 5 × the IC_30_ concentration of manzamine A, one enlarged vacuole was observed in more than 80% of the cells (*n* = 100). Testing vacuolar acidification by LysoSensor Green staining produced labeled vacuolar membranes in DMSO treated cells similar to the observed actions of the Vph1-GFP label. In both bafilomycin A1 and manzamine A treated cells, LysoSensor Green staining of the vacuole was completely absent suggesting a disruption of the pH gradient (Figure 2B). In summary, microscopic analysis of two v-ATPase dependent vacuolar parameters suggested similar effects of manzamine A to the established v-ATPase inhibitor bafilomycin A1.

### 2.3. Expression of V-ATPases in Pancreatic Cancer Cell Lines

**Figure 3:**
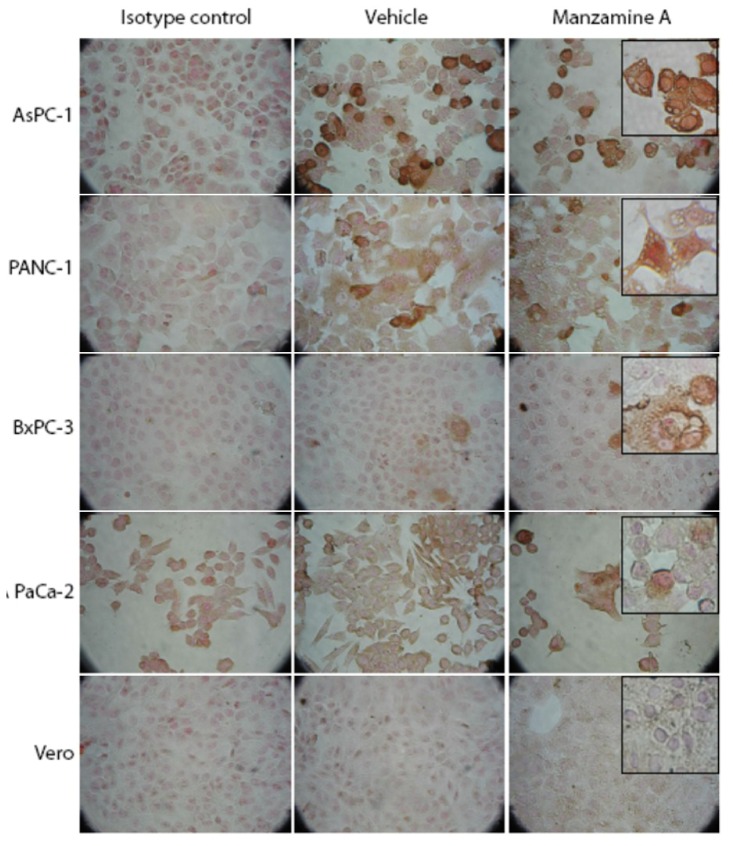
Manzamine A treatment had no effect on expression of v-ATPases in pancreatic cancer cells. AsPC-1, PANC-1, BxPC-3 and MIA PaCa-2 pancreatic cancer cells, as well as non-malignant Vero cells were treated with 10 µM manzamine A or methanol (vehicle control). 24 h later expression of v-ATPases was analyzed by immunocytochemistry with mouse monoclonal antibodies. Cells were analyzed at a magnification of 20× or 40× (inserts). One representative experiment out of three is shown.

Cells were seeded in 96-well plates and treated with manzamine A for 24 h. V-ATPase was detected by immunocytochemistry. V-ATPases were expressed in pancreatic cancer cell lines AsPC-1 and PANC-1 and to a less extent in BxPC-3 and MIAPaCa-2 (Figure 3). In contrast, the non-transformed cell line Vero showed only weak staining for v-ATPases. Manzamine A treatment had no effect on expression of v-ATPases.

### 2.4. Effect of Manzamine A on Proton Pump Activity of Mammalian V-ATPases

**Figure 4:**
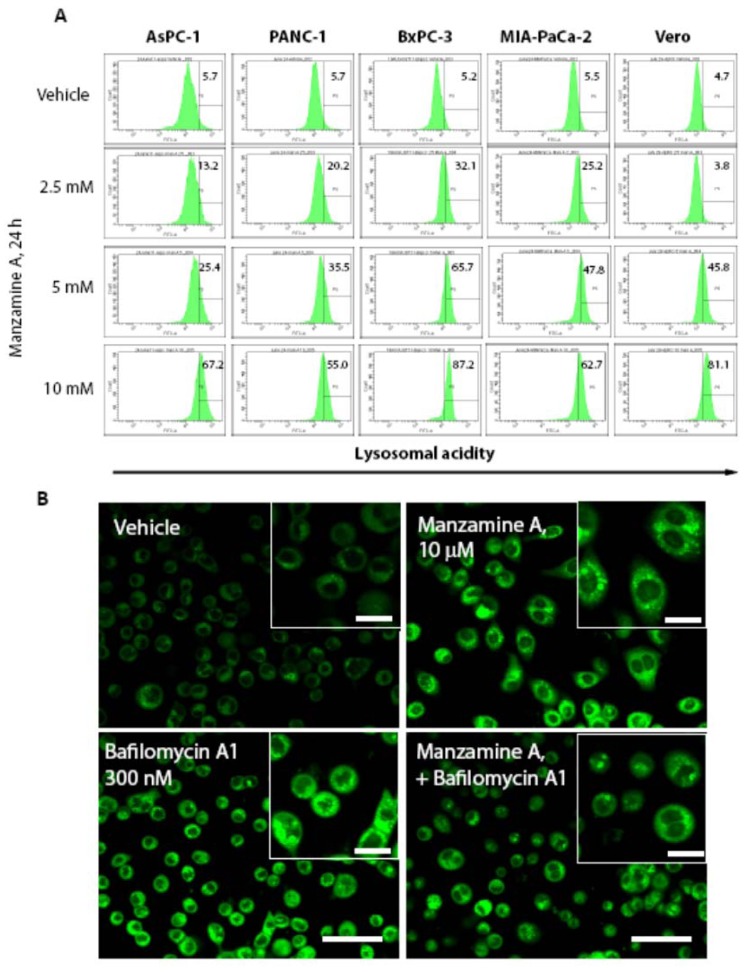
Manzamine A increases acidity in pancreatic cancer cells and non-malignant Vero cells. (**A**) AsPC-1, PANC-1, BxPC-3 and MIA PaCa-2 pancreatic cancer cells, as well as non-malignant Vero cells were treated with 2.5, 5, or 10 µM manzamine A or methanol (vehicle control). Twenty-four hours later cells were stained with Lysosensor green and analyzed by flow cytometry. Numbers in histograms indicate percentage (%) of cells with increased fluorescence intensity compared to vehicle control treated cells. One representative experiment out of three is shown. (**B**) AsPC-1 cells were treated for 2 h with 10 µM manzamine A or 300 nM bafilomycin A1 alone or in combination. Following treatment, cells were stained by Lysosensor green pH indicator followed by detection of acidic lysosomes by immunofluorescence microscopy at a magnification of 60×. One representative experiment out of three is shown. Size bar represents 150 µM in the main figures and 50 µM in the inserts.

As the chemogenomic profiling in yeast suggested that manzamine A is an uncoupler of v-ATPases, we investigated whether manzamine A inhibits v-ATPase activity in pancreatic cancer cells. Cells were treated for 24 h with manzamine A followed by staining with Lysosensor dye, which specifically stains acidic organelles such as lysosomes and shows a pH dependent increase of fluorescent intensity with increasing acidity. Interestingly, manzamine A treatment resulted in an dose dependent increase of fluorescence intensity, suggesting an increase in intralysosomal acidity and/or an accumulation of acidic organelles/lysosomes (Figure 4A). To clarify the effect of manzamine A on proton pump activity, its effect on v-ATPases was compared with the established inhibitor of v-ATPase, bafilomycin A1. AsPC-1 pancreatic cancer cells, which show the highest expression of v-ATPases, were treated with either manzamine A, bafilomycin A1 or a combination of both for 2 h. Treatment with bafilomycin A1 was restricted to 2 h as it is known that bafilomycin A1 has non-specific effects when administered for a prolonged period of time [27,28]. To directly compare both agents, manzamine A treatment was also for 2 h. Surprisingly, treatment with bafilomycin A1, similar to treatment with manzamine A resulted in an increase of fluorescence intensity following staining with Lysosensor (Figure 4B). Since bafilomycin A1 is an inhibitor of v-ATPases and as a result an inhibitor of autolysosome degradation, increase of acidity following staining with Lysosensor might be rather attributed to an accumulation of acidic autolysosomes following treatment with bafilomycin A1 and manzamine A. However, manzamine A treatment also induced an increase in acidity in nonmalignant Vero cells (Figure 4) which express low levels of v-ATPases (Figure 3), suggesting that manzamine A might have v-ATPase independent effects.

### 2.5. Effect of Manzamine A on Autophagy in Pancreatic Cancer Cells

Our results suggest that manzamine A treatment interferes with v-ATPase activity in pancreatic cancer cells and results in an accumulation of lysosomes/autolysosomes. Since lysosomes play an essential role in the autophagic pathway, our next aim was to elucidate the effect of manzamine A on autophagy.

**Figure 5:**
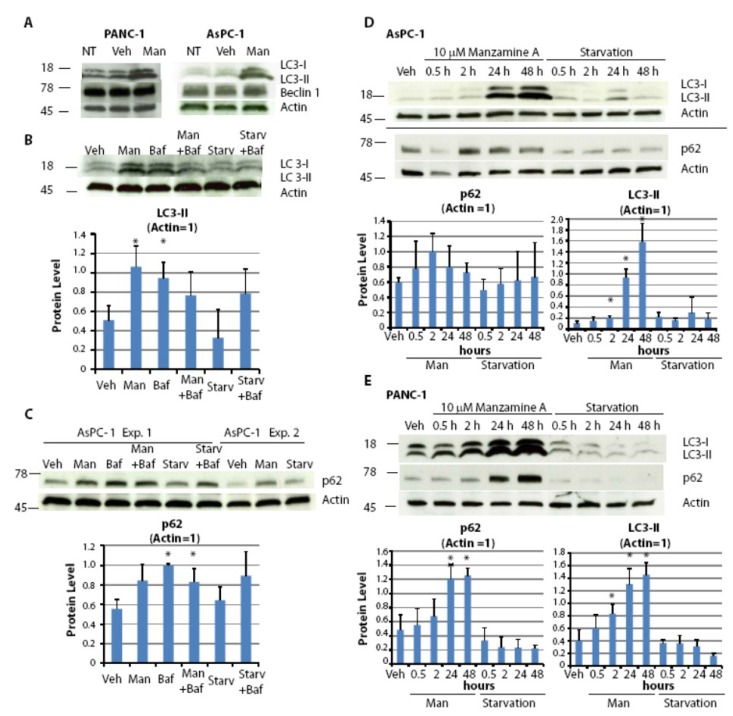
Manzamine A blocks the last step of the autophagic pathway. (**A**) AsPC-1 and PANC-1 cells were treated with media alone (NT), 10 µM manzamine A (Man) or methanol (vehicle control; Veh) for 24 h. Protein was extracted and LC3 and Beclin1 were detected by western blot analysis. (**B**) AsPC-1 cells, both grown normally or for 2 h in the absence of nutrients (absence of FBS and glutamine; Starv), were treated for 2 h with either 10 µM manzamine A (Man), 300 nM bafilomycin A1 (Baf) or a combination of both. Protein was extracted and LC3 was detected by western blot analysis. One representative experiment shown; graphs represent the mean ± standard deviation (SD) from five independent experiments. (**C**) AsPC-1 cells, grown normally or in the absence of nutrients, were treated for 2 h with either 10 µM manzamine A (Man), 300 nM bafilomycin A1 (Baf) or a combination of both. Protein was extracted and p62 was detected by western blot analysis. One representative experiment shown; graphs represent the mean ± SD from three independent experiments. (**D**) AsPC-1 and (**E**) PANC-1 cells were treated with 10 µM manzamine A (Man) for 0.5–48 h. Following protein extraction, LC3 and p62 were detected by western blot analysis. One representative experiment shown; graphs represent the mean ± SD from three independent experiments. An asterisk marks significant difference from vehicle control, *p* < 0.05 (*t* test). The numbers on the right side of the gels indicate molecular weight in KDa.

Pancreatic cell lines were treated with manzamine A for 24 h followed by protein extraction and western blot analysis. Manzamine A had no effect on expression of the autophagy gene Beclin1, but markedly induced expression of the autophagosome marker LC3-II (Figure 5A). An increase of LC3-II expression can be attributed to either an induction of autophagy or to an inhibition of autophagosome turnover, which is the last step of the autophagic pathway. To discriminate between these two distinct scenarios, we treated AsPC-1 pancreatic cancer cells with manzamine A in the presence or absence of the established v-ATPase inhibitor bafilomycin A1, which is known to inhibit fusion of lysosomes with autophagosomes. The treatment concentrations for manzamine A and bafilomycin A1 were chosen based on dose response experiments (data not shown, and our previous publication [17]). Manzamine A treatment increased LC3-II levels by itself, but did not change LC3-II levels in bafilomycin A1 treated cells compared to treatment with bafilomycin A1 alone (Figure 5B). These results suggest that manzamine A is a potential inhibitor of autophagy and acts at the level of autophagosome-lysosome fusion or autophagosome turnover. Furthermore, the effect of manzamine A on the levels of p62—a marker that inversely correlates with autophagic activity [29]—was tested. Manzamine A, similarly to bafilomycin A1, clearly induced an accumulation of p62 confirming an inhibition of autophagosome turnover (Figure 5C).

To further confirm that manzamine A inhibits autophagic flux, kinetic studies were performed following LC3-II expression. Similarly to p62, LC3-II is a substrate for autophagic degradation. An inhibitor of autophagosome turnover would result in a sustained accumulation of LC3-II, whereas an inducer of autophagy would result in a temporary increase of LC3-II followed by decrease in LC3-II levels due to its autophagic degradation. Pancreatic cancer cells were treated for times ranging from 30 min to 48 h with manzamine A followed by protein extraction and western blot analysis. Treatment with manzamine A resulted in a time dependent accumulation of LC3-II and confirms that manzamine A targets autophagosome turnover (Figure 5D,E). Treatment with manzamine A resulted in an accumulation of p62 in PANC-1 cells (Figure 5D) but only in a temporal increase of p62 in AsPC-1 cells (Figure 5E). This discrepancy could be due to the regulation of p62 by other signaling pathways [27,29]. Manzamine A had no effect on Beclin1 expression in AsPC-1 cells (data not shown).

In conclusion, treatment with manzamine A results in a sustained accumulation of the autophagosome marker LC3-II. Induction of LC3-II cannot be further induced in the presence of bafilomycin A1 suggesting an inhibition of autophagosome turnover following treatment with manzamine A. Consistently, treatment with manzamine A resulted in an accumulation of p62, demonstrating that manzamine A inhibits autophagy at the level of autophagosome/lysosome fusion or autophagosome turnover.

### 2.6. Discussion

Chemogenomic profiling showed a strong correlation of manzamine A with known uncouplers of v-ATPases in yeast. Microscopy in yeast showed marked changes in vacuolar numbers and acidity for cells treated with either manzamine A or the known V-ATPase inhibitor bafilomycin A1. Further evaluation of its effects on mammalian cells showed that while manzamine A had no effect on expression of v-ATPases in pancreatic cancer cells, it affects proton pump activity of v-ATPases. Manzamine A seems to have opposing effects on acidity in yeast *vs.* pancreatic cancer cells. In yeast cells, where v-ATPases are located in vacuolar membranes, inhibition of v-ATPase activity by bafilomycin A1 or manzamine A resulted in a decrease in vacuolar acidity. However, in pancreatic cancer cells, treatment with manzamine A resulted in an increase of acidity. Since the same result was observed following treatment with the established v-ATPase inhibitor bafilomycin A1, the perceived increase in acidity might reflect an accumulation of acidic organelles within pancreatic cancer cells rather than an increase of acidity within lysosomes. This is also consistent with our observation that treatment of pancreatic cancer cells with manzamine A blocks the last step of the autophagic pathway resulting in an accumulation of autophagosomes, acidic lysosomes, and /or acidic autolysosomes, similar to that seen with bafilomycin A1 treatment. In support of our findings, previous publications have shown that inhibitors of autophagosome turnover, such as chloroquine and hydroxychloroquine, induce autophagosome accumulation accompanied by an increase in the acidic vacuolar compartment [30,31]. V-ATPases are also present in the plasma membrane of pancreatic cancer cells [1], where they pump protons to the exterior. Thus, inhibition of v-ATPases with manzamine A could also result in an accumulation of protons in the cytoplasm and thus an increase of cytosolic acidity. One could speculate an increased pumping/overshooting of protons into the lysosomal lumen as a protective mechanism that counteracts an accumulation of protons in the cytoplasma caused by manzamine A. In fungi this compensatory effect could be missing or the thick fungal cell wall might change kinetics of proton pumping. Moreover, a recent publication supports our data and shows an increase of acidity in the pancreatic cancer cell line AsPC-1 following treatment with the proton pump inhibitor omeprazole [32], which has been suggested to inhibit v-ATPases [33].

**Figure 6:**
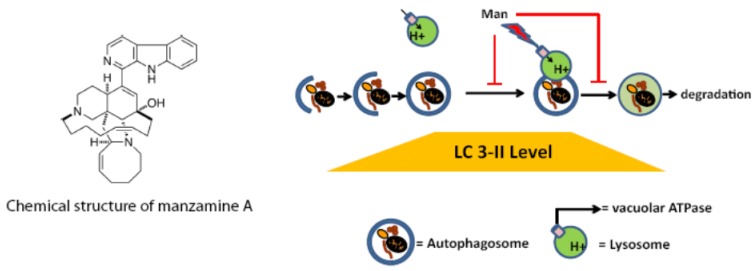
Structure of manzamine A and illustration of its potential mechanism of action. Manzamine A interferes with proton pump activity of v-ATPases in pancreatic cancer cells, induces an increase in the acidic vacuolar/lysosomal compartment, and inhibits autophagy at the level of autophagosome–lysosome fusion and/or autophagosome turnover.

The effects of manzamine A on v-ATPases suggested a potential effect on the autophagic pathway in pancreatic cancer cells. At first we aimed to evaluate the effects of manzamine A on Beclin1, which is an inducer of autophagy and was suggested to regulate autophagy and membrane trafficking in several physiological and pathological processes [34,35]. Beclin1 expression was already prominent in untreated pancreatic cancer cells cultured in presence of nutrients (FBS/glutamine) suggesting a high level of basal autophagy, in accordance with a recent publication [36]. Manzamine A had no effect on expression of Beclin1 indicating that it does not further induce autophagy in pancreatic cancer cells. However, we demonstrated that treatment with manzamine A results in a sustained accumulation of the autophagosome marker LC3-II in AsPC-1 and PANC-1 cells, as well as an accumulation of p62 in PANC-1 cells, indicating a blockade of the autophagosome turnover in pancreatic cancer cell lines. p62 expression increased only temporarily in AsPC-1 cells treated with manzamine A for 2 h, and p62 levels returned to the same level as in vehicle controls when treated for 24 or 48 h. Because p62 is regulated at the transcriptional level by oxidative stress and by the Ras oncogene, detection of LC3-II is a better marker to evaluate autophagosome turnover [27]. While manzamine A increased the LC3-II level by itself, it did not change LC3-II levels in bafilomycin A1 treated cells compared to treatment with bafilomycin A1 alone. Altogether, these results suggest that manzamine A blocks autophagy at the level of autophagosone-lysosome fusion and/or autolysosome degradation. The effects of manzamine A in autophagy are illustrated in Figure 6. The accumulation of autophagosomes and/or autolysosomes caused by manzamine A could potentially also have an effect on macropinocytosis, a process by which Ras driven tumors obtain glutamine for growth [8].

Manzamine A sensitizes the pancreatic cancer cell line AsPC-1 to TRAIL induced apoptosis [17]. The accumulation of autophagosomes observed following treatment with manzamine A might explain its pro-apoptotic effects. Previous reports have shown that an accumulation of autophagic vacuoles resulting from inhibition of v-ATPases and/or autophagy are linked to enhanced cell death triggered by lysosomal dysfunction or lysosomal membrane permeabilization leading to the release of lysosomal enzymes that cause cell death [30,37,38,39,40].

Autophagy can contribute to chemotherapeutic resistance [26,41] and has been shown to support pancreatic cancer cell survival [7]. Moreover, autophagy was shown to be enhanced in pancreatic cancer stem cells [42] and essential for tumor growth suggesting that pancreatic cancer is specifically prone to agents that block this pathway [7,36]. Therefore, manzamine A may be a promising compound to target pancreatic cancer as well as other tumor entities. While bafilomycin A1 also targets v-ATPases and inhibits autophagy, it is not well tolerated *in vivo* [43]. In contrast, manzamine A was well tolerated in *in vivo* studies for malaria [16].

## 3. Experimental Section

### 3.1. Reagents

Manzamine A was obtained from the Harbor Branch Oceanographic Institution pure compound library. The material was originally isolated from a sponge of the genus *Haliclona* as described in US patent # 4,895,854. The manzamine A stock solution was at a concentration of 1 mg/mL in methanol. Bafilomycin A1 was purchased from Calbiochem/EMD Biosciences, San Diego, CA, USA.

### 3.2. Cell Culture and Treatment

The human pancreatic cancer cell lines PANC-1 (CRL-1469), AsPC-1 (CRL-1682), MIA PaCa-2 (CRL-1420), BxPC3 (CRL-1687) as well as the control cell line Vero (CCL-81, African-green monkey kidney), were obtained from ATCC and cultured as described previously [44,45]. Cells were expanded, aliquotted and frozen. Once thawed, cells were only maintained in culture for 10 weeks, when a new aliquot was thawed. No authentication was performed in our labs. For starvation experiments, cells were grown in RPMI medium in the absence of FBS (HyClone Laboratories Inc, Logan, UT, USA) and with a final l-glutamine concentration of 2 mM. If not indicated otherwise, cancer cells were treated with 10 µM manzamine A and/or 300 nM bafilomycin A1.

### 3.3. Chemogenomic Profiling

The potency of test substances was determined using wild-type *S. cerevisiae* BY4743. OD600 values of exponentially growing *S. cerevisiae* cultures in rich medium were recorded with a robotic system. Twelve point serial dilutions were assayed in 96 well plates with a reaction volume of 150 µL. Solutions containing dimethyl sulfoxide (DMSO) were normalized to 2%. IC30 values were calculated using logistic regression curve fits generated by TIBCO Spotfire v3.2.1 TIBCO Software Inc.

The Saccharomyces haploinsufficiency profiling (HIP) and homozygous profiling (HOP) and microarray analysis was performed as previously published [20]. The genome wide hetero- and homozygous deletion libraries of *S. cerevisiae* strains were purchased (OpenBiosystems, Cat # YSC1056 and YSC1055), and pools were constructed as previously published [20]. Each test substance was assayed in duplicate, at its IC_30_ concentration, and relative abundance of all strains in the pools compared to eight no drug control samples. Specific HIP HOP assay adaptations concerning starting culture density, reaction culture volume, dilution scheme, and experimental controls are described in Hoepfner *et al.*, 2013 [46]. For the experimental analysis we used the same computation of normalized tag intensities, outlier masking and saturation correction as published [20]. Sensitivity was computed as Median Absolute Deviation Logarithmic (MADL) score for each compound/concentration combination, then gene-wise z-scores (across all experiments) which are based on a robust parametric estimation of gene variability allowing for up to 15% outliers were computed as described in detail in Hoepfner *et al.*, 2013 [46]. Similarity of the obtained HOP profiles was compared by calculating the Pearson correlation coefficients of a compound HOP profile with all other compound profiles. In order to assess the significance of the correlation coefficients we transform them by the Fisherz’ transformation and fitted a normal distribution to the transformed values. This empirical normal distribution allowed us to assign a p-value to each correlation coefficient. The (negative) logarithm of the false discovery rate (FDR) corrected p-value is then displayed.

### 3.4. Yeast Fluorescence Microscopy

Images were collected using an Axiovert 200 m microscope (Carl Zeiss, Feldbach Switzerland), with an AxioCam MRm monochrome CCD camera (Carl Zeiss, Feldbach Switzerland) and an AxioVision 4.6 software package (Carl Zeiss, Feldbach Switzerland). For LysoSensor Green staining (Cat # L-7534, Molecular Probes, Eugene, OR, USA), log phase cultures were pre-incubated at 5 × IC30 compound for 60 min in YPD medium. Pictures were taken within 1 h of labeling using the FITC filter set. For analysis of GFP-tagged cells, log phase cultures in YPD medium were incubated at 5 × IC30 compound for 60 min. Three fluorescent images per channel along the z-axis were acquired. Selection of representative sections and cells and assembly of panels was done in Photoshop 7 (Adobe Systems).

### 3.5. Immunocytochemistry

Cells were seeded in 96 well plates and treated with manzamine A or vehicle control (methanol). 24 h post-treatment v-ATPase was detected by immunocytochemistry. Briefly, cells were fixed with pre-warmed 4% paraformaldehyde at 37 °C and were permeabilized with ice cold methanol. Cells were incubated with 1% bovine serum albumin in PBS containing 0.2% Tween for 30 min followed by incubation with monoclonal v-ATPase antibody (ATP6V0D1, Abcam, Cambridge, MA, USA) or isotype antibody (negative control, Jackson ImmunoResearch, West Grove, PA, USA). Following incubation with anti-mouse-IgG-HRP secondary antibody (Jackson ImmunoResearch, West Grove, PA, USA), cells were incubated with 3,3′ diaminobenzidine, fixed with Slow Fade Gold antifade Reagent (Molecular Probes, Eugene, OR, USA) and analyzed by microscopy (Olympus IX71, Center Valley, PA, USA).

### 3.6. Lysosensor Assay-Detection of Acidic Vacuoles in Pancreatic Cancer Cells

To measure pH of acidic organelles, cells were seeded in 6-well plates, followed by treatment with 2.5, 5, or 10 µM manzamine A for 24 h. Cells were collected and incubated with 1 µM Lysosensor DND-189 (Cat # L-7535, Molecular Probes, Eugene, OR, USA) for 1 min on ice followed by flow cytometric analysis. For detection of acidic vacuoles/lysosomes by immunofluorescence, cells were seeded on poly-lysine coated glass bottom dishes (MatTek Corporation, Ashland, MA, USA) and were treated with manzamine A, bafilomycin A1, or both agents in combination for 2 h. Following treatment, cells were incubated with Lysosensor DND-189 for 30 min and subsequently analyzed by immunofluorescence microscopy (Olympus FV 300, Center Valley, PA, USA) using FLUOVIEW software.

### 3.7. Western Blotting

AsPC-1 cells were treated with manzamine A for times ranging from 0.5 to 48 h, in the presence or absence of bafilomycin A1. Western blotting was performed as recently described [17]. The antibodies used were anti-LC3 (Cat # 3868), anti-p62 (Cat # 8025) and anti-Beclin1 (Cat # 3495) (Cell Signaling Technologies, Beverly, MA, USA) and anti-β actin (Sigma Chemical Co., St. Louis, MO, USA). Peroxidase-conjugated anti-rabbit IgG secondary antibody was obtained from Jackson ImmunoResearch (West Grove, PA, USA). ImageJ software was used for protein quantification. Statistical analysis of the data sets to determine mean and standard deviation was performed using Microsoft Excel. Data sets were compared using the Student’s *T* Test. A *p* value ≤ 0.05 was considered significant.

## 4. Conclusions

Our results indicate that manzamine A interferes with proton pump activity of v-ATPases in yeast and pancreatic cancer cells. In pancreatic cancer cells, manzamine A treatment resulted in a blockade of the autophagic pathway at the level of lysosome-autophagosome fusion or autophagosome turnover. Since autophagy is essential for pancreatic tumor growth and chemoresistance, targeting this pathway with manzamine A suggests a promising treatment strategy for PDAC.
