# Correction: Kallifatidis, G. *et al.* The Marine Natural Product Manzamine A Targets Vacuolar ATPases and Inhibits Autophagy in Pancreatic Cancer Cells. *Mar. Drugs* 2013, *11*, 3500–3516

**DOI:** 10.3390/md12042305

**Published:** 2014-04-21

**Authors:** Georgios Kallifatidis, Dominic Hoepfner, Tiphaine Jaeg, Esther A. Guzmán, Amy E. Wright

**Affiliations:** 1 Marine Biomedical and Biotechnology Research Program, Harbor Branch Oceanographic Institute, Florida Atlantic University, 5600 US 1 North, Fort Pierce, FL 34946, USA; E-Mails: geokallifa@aol.com (G.K.); awrigh33@hboi.fau.edu (A.E.W.); 2 Novartis Institutes for BioMedical Research, Developmental & Molecular Pathways, Novartis Pharma AG, WSJ-355.1.051.21, Fabrikstrasse 22, Basel CH-4056, Switzerland; E-Mails: dominic.hoepfner@novartis.com (D.H.); tiphaine.jaeg@wanadoo.fr (T.J.)

We found two errors in our previous published paper [1]. Figure 4A has a mistake in the units in the labels, where it shows mM instead of micromolar (μM). A correctly labeled Figure 4A ensues. In Figure 2 and Figure 4, the size bar scale is micrometers (μm). We apologize for the inconvenience caused to our readers. 

**Figure 2:**
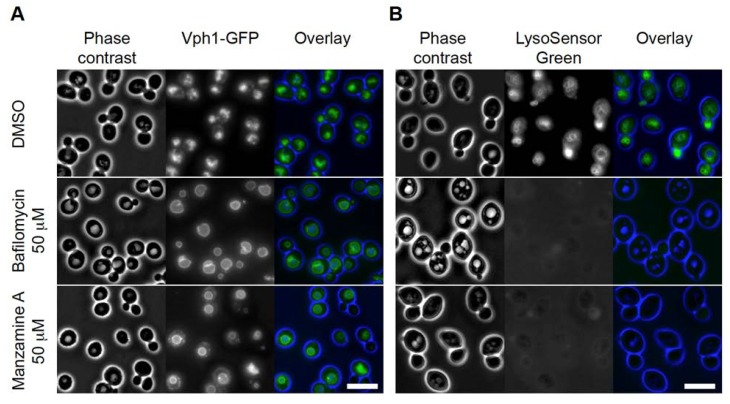
Manzamine A affects vacuolar morphology and acidification in yeast, similar to bafilomycin. (**A**) Vacuolar morphology analysis using Vph1-GFP (a v-ATPase V0 domain) as marker. DMSO treated cells showed clusters of small vacuoles. In bafilomycin A1 treated cells one large vacuole was detected in almost all cells. Manzamine A treated cells displayed a few enlarged vacuoles similar to the situation observed in bafilomycin A1 treated cells. (**B**) Vacuolar acidification analysis using LysoSensor Green as marker. DMSO treated cells show staining of the vacuolar membranes. Treatment of cells with bafilomycin A1 or manzamine A results in abolishment of detectable vacuolar staining. Size bar represents 5 µm.

**Figure 4:**
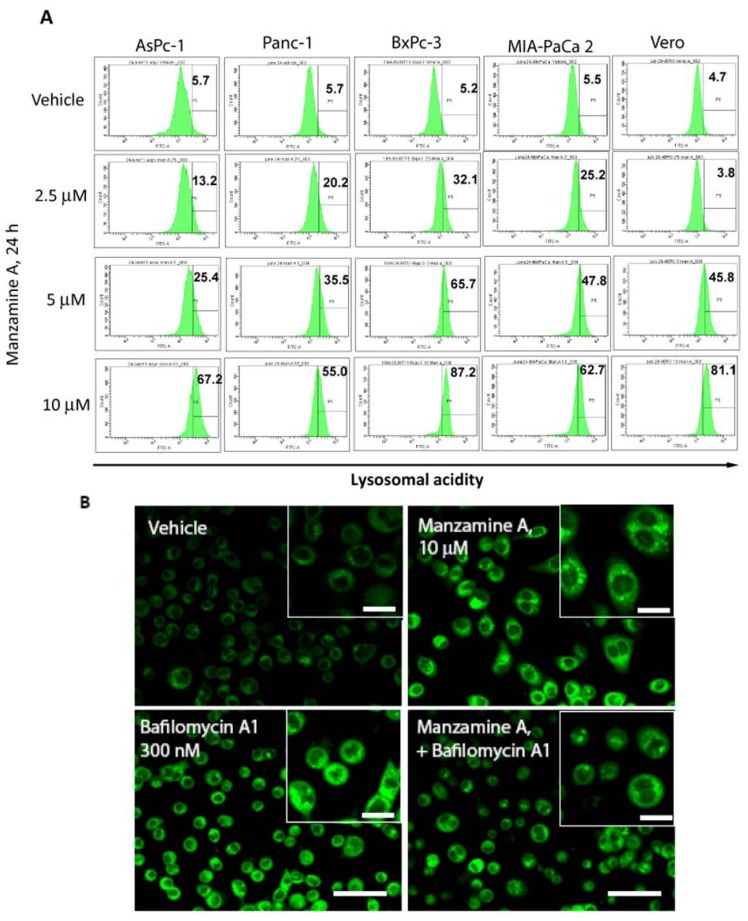
Manzamine A increases acidity in pancreatic cancer cells and non-malignant Vero cells. (**A**) AsPC-1, PANC-1, BxPC-3 and MIA PaCa-2 pancreatic cancer cells, as well as non-malignant Vero cells were treated with 2.5, 5, or 10 μM manzamine A or methanol (vehicle control). Twenty-four hours later cells were stained with Lysosensor green and analyzed by flow cytometry. Numbers in histograms indicate percentage (%) of cells with increased fluorescence intensity compared to vehicle control treated cells. One representative experiment out of three is shown. (**B**) AsPC-1 cells were treated for 2 h with 10 µM manzamine A or 300 nM bafilomycin A1 alone or in combination. Following treatment, cells were stained by Lysosensor green pH indicator followed by detection of acidic lysosomes by immunofluorescence microscopy at a magnification of 60×. One representative experiment out of three is shown. Size bar represents 150 µm in the main figures and 50 µm in the inserts.

## References

[1] Kallifatidis G., Hoepfner D., Jaeg T., Guzmán E.A., Wright A.E. (2013). The Marine Natural Product Manzamine A Targets Vacuolar ATPases and Inhibits Autophagy in Pancreatic Cancer Cells. Mar. Drugs.

